# Three decades of neurocysticercosis mandatory notification in a Brazilian endemic region: clinical evolution and severity patterns

**DOI:** 10.1007/s10096-026-05555-9

**Published:** 2026-07-07

**Authors:** Tissiana Marques de Haes, Thales Pardini Fagundes, Trajano Aguiar Pires Gonçalves, Osvaldo Massaiti Takayanagui

**Affiliations:** 1 Department of Neurosciences and Behavioral Sciences Ribeirão Preto School of Medicine, University of São Paulo, Av. Bandeirantes, 3900, Campus USP, Ribeirão Preto, SP 14049-900 Brazil; 2 Department of Neuro-Oncology Hospital de Amor de Barretos São Paulo, São Paulo, Brazil

**Keywords:** Neurocysticercosis, *Taenia solium*, Intracranial hypertension, Hydrocephalus, Ventriculoperitoneal shunt, Arachnoiditis, Epilepsies, partial, Magnetic resonance imaging, Mandatory reporting, Incidence

## Abstract

**Background:**

Neurocysticercosis (NCC) is the leading cause of late-onset epilepsy in endemic regions, yet long-term data on severe forms remain limited. This study analyzed temporal trends in severe NCC over three decades following mandatory notification in a high-incidence region of Brazil.

**Methods:**

This retrospective population-based study included Neurocysticercosis cases reported from January 1995 to September 2024 in a high-incidence endemic area of São Paulo State, Brazil. Confirmed NCC cases were reclassified as intracranial hypertension syndrome (IHS) or other clinical presentations (epileptic, meningeal, asymptomatic). Clinical, neuroimaging, and cerebrospinal fluid data were analyzed. Multivariable logistic regression identified factors associated with severe disease.

**Results:**

Of 518 reported cases, 465 confirmed NCC cases were included. In the first decade (1991–2000, *n* = 262), 13.4% (35/262) were severe. Between these periods (2001–2010, *n* = 125), a steady decline in total incidence was observed. In the most recent period (2011–2020, *n* = 78), the proportion of severe cases rose to 65.4% (51/78). Meningeal manifestations were associated with increased severity risk (OR = 2.99; 95%CI: 1.81–4.94), whereas epileptic (OR = 0.30; 95%CI: 0.18–0.48) and asymptomatic presentations (OR = 0.21; 95%CI: 0.11–0.40) were protective. Neurosurgical procedures were required in 79.4% of severe cases.

**Conclusions:**

Despite declining overall incidence, the increasing proportion of severe NCC (intracranial hypertension) likely reflects late-stage diagnoses of long-standing infections that remained asymptomatic for decades. This trend suggests a ‘catch-up’ effect in detecting complex cases through improved neuroimaging, even as primary prevention programs successfully reduce new infections. .

## Introduction

Cysticercosis, caused by the larval form of *Taenia solium*, represents a serious public health problem in developing countries where poor sanitary conditions perpetuate its dissemination [1]. Neurocysticercosis (NCC) affects the central nervous system [2] and is the major cause of late-onset epilepsy in endemic areas. The global prevalence remains uncertain due to heterogeneous epidemiological data, though a recent systematic review estimated that NCC is the etiology of epilepsy in 29% of patients in endemic regions [3]. Most infected individuals are between 20 and 50 years old, with multifaceted social and economic impacts [4].

Clinical manifestations depend on morphological type, number, location, developmental stage, and host immunological reactions [5–9]. This results in pleomorphic presentations with multiple neurological signs and symptoms, without pathognomonic features [9–11]. Intraparenchymal NCC typically has good prognosis, with patients having few cysts often remaining asymptomatic or presenting epileptic seizures. However, those with massive infestation may present intracranial hypertension syndrome, refractory seizures, psychiatric disorders, and cognitive deficits [12, 13].

Racemose NCC represents the most severe form, with prolonged incubation (10–25 years) characterized by cystic proliferation and growth with invasion of contiguous spaces. This generates mass effect and, combined with host immunological response, causes arachnoiditis, hydrocephalus, infarcts, and inflammatory manifestations. Racemose NCC represents the most lethal form, with mortality rates 15 times higher than intraparenchymal forms [14]. Epileptic seizures occur in 70–90% of patients and represent the most frequent manifestation [14, 15]. In the ventricular system, cysticerci cause intracranial hypertension secondary to hydrocephalus, while subarachnoid cysts may increase in volume causing intracranial hypertension with focal signs. Racemose cysticercosis in basal cisterns causes intense inflammatory reaction, progressive leptomeningeal thickening, and cerebrospinal fluid obstruction in approximately 60% of cases [16].

Diagnosis relies on cerebrospinal fluid examinations, neuroimaging, and antibody detection. The WHO emphasizes neuroimaging importance, recommending both computed tomography and magnetic resonance imaging for suspected cases [17]. Diagnostic criteria frameworks were developed in 1996 [18], revised in 2001 [19] and 2017 [20] , integrating different information categories with defined certainty degrees. Treatment involves managing acute symptoms first, followed by individualized antiparasitic therapy [21, 22]. Albendazole is the drug of choice for symptomatic patients with viable cerebral cysts [22, 23], with corticosteroids initiated before and maintained during antiparasitic treatment [24, 25]. However, antiparasitic therapy remains marked by intense controversy regarding clinical benefit and safety [24, 25]. Surgical treatment is restricted to cerebrospinal fluid shunt placement and isolated cyst removal cases [26, 27].

A high-incidence region in interior Brazil implemented mandatory notification in 1992 as part of the Taeniasis/Cysticercosis Prevention Program [27]. This study aimed to analyze clinical and laboratory characteristics of NCC cases over three decades, evaluate temporal evolution of ISH forms, and assess prevention program success in an endemic area.

## Methods

This retrospective, observational, document-based study examined cysticercosis cases from January 1995 to September 2024 in a high-incidence region of interior São Paulo State, Brazil, constituting three decades following mandatory notification implementation. The study included all patients diagnosed with neurocysticercosis in regional healthcare services, including residents from surrounding municipalities receiving care at the referral center. Inclusion criteria comprised: (1) confirmed neurocysticercosis diagnosis based on neuroimaging findings and/or cerebrospinal fluid analysis, (2) complete medical records available for review, and (3) cases reported through the mandatory notification system. Exclusion criteria included: (1) suspected but unconfirmed cases, (2) incomplete medical records preventing clinical classification, and (3) cases diagnosed exclusively by necropsy without sufficient clinical data for severity assessment (22 cases excluded from clinical analysis but included in epidemiological counts). Cases were identified through notification forms and medical records from the Clinical Hospital of Ribeirão Preto School of Medicine, University of São Paulo.

Clinical characteristics and laboratory data from 465 reported cases were evaluated. Cases were reclassified into IHS presentations (29.2% of cases) and other clinical presentations (70.8% of cases, including epileptic and meningeal presentations, as well as asymptomatic cases). Incidence rates were calculated annually for the study region and surrounding municipalities in the Regional Health Department XIII, with analysis comparing rates between groups and evaluating temporal trends.

Neuroimaging findings, cerebrospinal fluid alterations, treatments performed, and clinical complications were analyzed. Missing data were handled using complete case analysis, with patients excluded only if essential variables for the specific analysis were unavailable. The proportion of missing data was minimal (< 5%) for key variables, and missing data patterns were assessed to ensure they were missing at random. Multivariable logistic regression identified factors independently associated with severe disease, with statistical significance set at *p* < 0.05.

### Standard protocol approvals, registrations, and patient consents

The study was approved by the Research Ethics Committee of the Clinical Hospital of Ribeirão Preto School of Medicine, University of São Paulo. Written informed consent was waived due to the retrospective nature of the study.

### Diagnostic criteria and variable definitions

Neurocysticercosis diagnosis was established using the revised diagnostic criteria proposed by Del Brutto et al. (2017), integrating neuroimaging findings, cerebrospinal fluid analysis, and clinical manifestations. Intracranial Hypertension Syndrome (IHS) was defined as cases presenting with increased intracranial pressure, characterized by headache, nausea, vomiting, papilledema, altered consciousness, or hydrocephalus documented by neuroimaging. Other clinical presentations included epileptic presentations (seizures without intracranial hypertension), meningeal presentations (meningeal signs without intracranial hypertension), and asymptomatic cases (incidental neuroimaging findings without clinical manifestations).

### Sample size justification

This study encompassed all neurocysticercosis cases reported through the mandatory notification system during the 30-year period (1995–2024), constituting a population-based cohort study. No formal sample size calculation was performed as this represented a comprehensive analysis of all available cases in the endemic region during the study period. The final sample of 465 confirmed cases provided sufficient statistical power (> 80%) to detect clinically meaningful differences between ISH and other clinical presentations, considering the observed effect sizes and the 29.2% prevalence of ISH forms.

### Data availability

Anonymized data will be made available to qualified investigators upon reasonable request to the corresponding author.

## Results

From January 1995 to September 2024, 518 new cysticercosis cases were reported in this high-incidence region, including 197 residents from the study area, 132 from other Regional Health Department XIII municipalities, and 189 from other locations. Of these reported cases, 465 patients with confirmed NCC diagnosis were included for clinical form analysis, with 136 (29.2%) presented IHS and 329 (70.8%) other clinical presentation**s**. The absolute risk of developing severe disease was 29.2% (95% CI: 25.1%-33.6%) among all confirmed NCC cases in this endemic region.

**Fig. 1:**
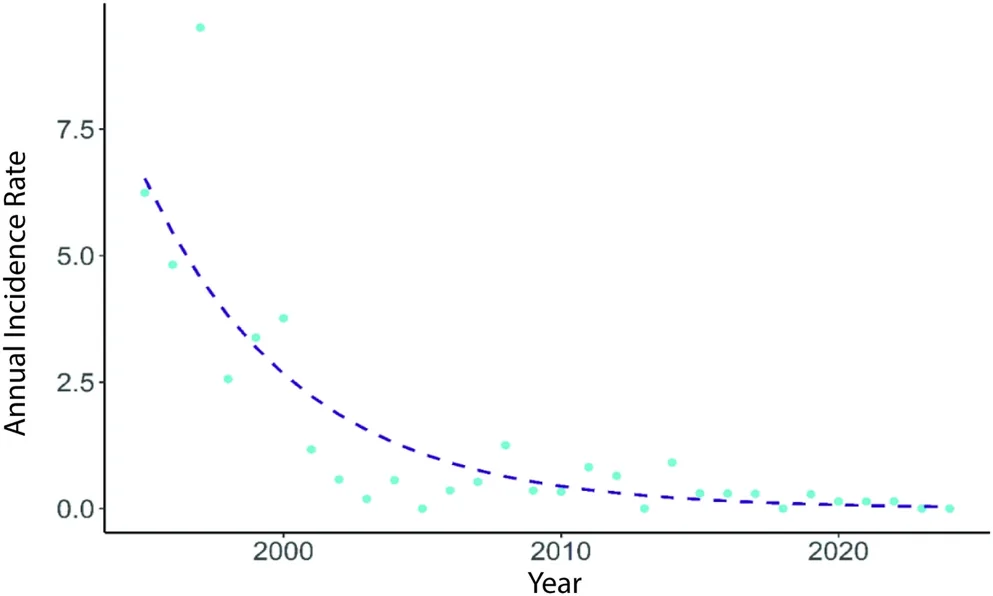
Temporal evolution of neurocysticercosis incidence in Ribeirão Preto. Annual incidence rates of neurocysticercosis cases per 100,000 inhabitants in Ribeirão Preto from 1995 to 2024, demonstrating significant decline over three decades following implementation of the Taeniasis/Cysticercosis prevention program. The graph shows more pronounced reduction compared to surrounding municipalities in regional health department XIII, supporting the effectiveness of mandatory notification and prevention measures implemented exclusively in Ribeirão Preto

A significant decline in NCC incidence rates occurred from 1995 to 2024, with the decline more pronounced in the primary study region compared to surrounding municipalities, suggesting success of the Prevention Program implemented in this endemic area (Table 1; Fig. 1). Concurrently, a progressive increase in the proportion of patients with IHS occurred over three decades, from 13.4% in the first decade (1991–2000; 35/262) to 65.4% in the last decade (2011–2020; 51/78) (Cochran-Armitage trend test; chi-square 30.99; *p* < 0.0001), with significant trends observed throughout the period and rate convergence in 2011–2020.

**Table 1:** Demographic profile and temporal evolution of neurocysticercosis patients by disease presentation

Characteristics	Other clinical presentations (*n* = 329)	Intracranial hypertension syndrome (*n* = 136)	*P* Value
Demographic Characteristics			
Age (years)*	43.2 (17.8)	45.3 (13.4)	0.222
Age Groups (years), n (%)			0.182
≤ 20	42 (12.8)	12 (8.8)	
21–40	115 (35.0)	41 (30.1)	
41–60	127 (38.6)	63 (46.3)	
> 60	45 (13.6)	20 (14.8)	
Male sex, n (%)	169 (51.4)	77 (56.6)	0.353
Geographic Origin, n (%)			0.048
Primary study region	131 (39.8)	36 (26.5)	
Regional Health Dept. XIII	99 (30.1)	32 (23.5)	
Other locations	99 (30.1)	68 (50.0)	
Diagnostic Period, n (%)			< 0.001
1991–2000	227 (69.0)	35 (25.7)	
2001–2010	75 (22.8)	50 (36.8)	
2011–2020	27 (8.2)	51 (37.5)	

Comparison of demographic characteristics and temporal distribution between IHS form (*n* = 136) and other clinical presentation forms (*n* = 329) of neurocysticercosis. Data presented as mean (standard deviation) for continuous variables and n (%) for categorical variables. Statistical significance assessed using Student’s t-test for continuous variables and chi-square or Fisher’s exact test for categorical variables. Notable findings include significant differences in geographic origin (*p* = 0.048) and diagnostic period (*p* < 0.001), with ISH forms more frequently originating from locations outside the Regional Health Department XIII and diagnosed in more recent periods, suggesting improved detection capabilities for complex cases

*Data presented as mean (standard deviation) for continuous variables and n (%) for categorical variables

*P* values calculated using Student’s t-test for continuous variables and chi-square or Fisher’s exact test for categorical variables

Comparison of demographic characteristics and temporal distribution between IHS form (*n* = 136) and other clinical presentation (*n* = 329) of neurocysticercosis. Notable findings include significant differences in geographic origin (*p* = 0.048) and diagnostic period (*p* < 0.001), with IHS forms more frequently originating from locations outside the Regional Health Department XIII and diagnosed in more recent periods, suggesting improved detection capabilities for complex cases

### Clinical manifestations

For detailed clinical manifestations analysis, 443 patients were considered (465 minus 22 patients diagnosed through necropsy). In initial evaluation, 181 patients were categorized as ISH forms; however, through proposed reclassification allocating only intracranial hypertension syndrome patients to ISH form, 45 patients were reclassified, leaving 136 in the final ISH form group. Clinical manifestations presented significant differences between groups (Table 2).

**Table 2:** Clinical manifestations of neurocysticercosis patients by disease presentation

Clinical characteristics	Other clinical presentation (*n* = 307)	Intracranial hypertension syndrome (*n* = 136)	*P* Value
Clinical Manifestations			
Epileptic manifestations, n (%)	239 (77.8)	61 (44.9)	< 0.001
Meningeal manifestations, n (%)	49 (15.9)	65 (47.7)	< 0.001
Asymptomatic presentation, n (%)	43 (14.0)	0 (0)	< 0.001
Neuroimaging Findings			
Viable cysts, n (%)	99 (32.2)	105 (77.2)	< 0.001
Calcified lesions, n (%)	188 (61.2)	37 (27.2)	< 0.001
Hydrocephalus, n (%)	0 (0)	75 (55.1)	< 0.001
Cerebrospinal Fluid			
Pleocytosis, n (%)	33 (10.7)	49 (36.0)	< 0.001
Positive ELISA, n (%)	13 (4.2)	21 (15.4)	< 0.001
Treatment and Procedures			
Antiparasitic therapy, n (%)	156 (50.8)	89 (65.4)	0.004
Neurosurgery for cyst removal, n (%)	7 (2.3)	27 (19.9)	< 0.001
Ventriculoperitoneal shunt, n (%)	1 (0.3)	74 (54.4)	< 0.001

Distribution of main clinical manifestations stratified by disease severity among 443 patients (excluding 22 cases diagnosed through necropsy). The ISH form group showed significantly higher frequency of meningeal manifestations (47.7% vs. 15.9%, *p* < 0.001) and lower occurrence of epileptic manifestations (44.9% vs. 77.8%, *p* < 0.001) compared to other clinical presentations. Asymptomatic presentation occurred exclusively in the other clinical presentations group (14.0%). These patterns support the clinical classification system and highlight distinct phenotypic differences between IHS and other clinical manifestations of neurocysticercosis Data presented as *n* (%)

Data presented as *n* (%)

Distribution of main clinical manifestations stratified by disease severity among 443 patients (excluding 22 cases diagnosed through necropsy). The ISH form group showed significantly higher frequency of meningeal manifestations (47.7% vs. 15.9%, *p* < 0.001) and lower occurrence of epileptic manifestations (44.9% vs. 77.8%, *p* < 0.001) compared to other clinical presentations. Asymptomatic presentation occurred exclusively in the other clinical presentations group (14.0%). These patterns support the clinical classification system and demonstrate distinct phenotypes between severe and non-severe neurocysticercosis presentations

**Fig. 2:**
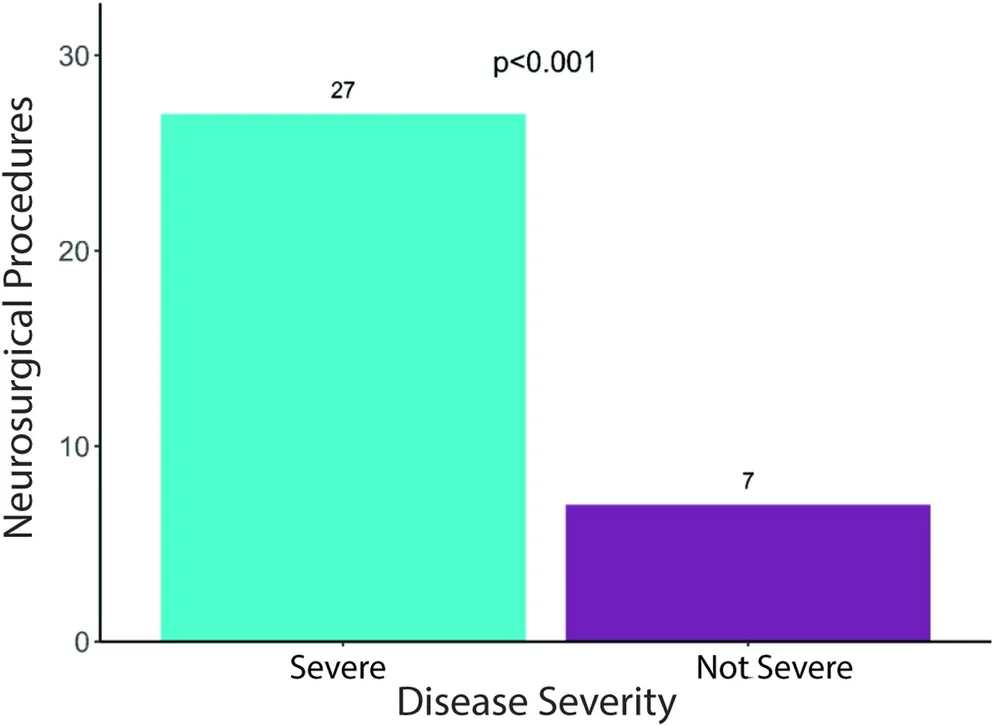
Neurosurgical procedures by disease severity. Distribution of patients who underwent neurosurgery for cyst removal according to the presence of IHS. Of the 34 patients (7.6% of the sample) who underwent neurosurgical procedures, 27 (79.4%) presented with IHS, demonstrating a strong association between this specific clinical phenotype and the need for surgical intervention. This pattern reflects the predominance of extraparenchymal cysts and hydrocephalus in IHS presentations requiring surgical management

The severe group showed higher frequency of meningeal manifestations (47.7% vs. 15.9%) and lower occurrence of epileptic manifestations (44.9% vs. 77.8%), with some cases presenting overlap of multiple clinical manifestations. Asymptomatic manifestation occurred in 14.0% of the non-severe group. Multivariable analysis identified independent protective factors against IHS: epileptic manifestations (OR = 0.295; 95%CI: 0.182–0.477; *p* < 0.001) and asymptomatic presentation (OR = 0.212; 95%CI: 0.111–0.404; *p* < 0.001), while meningeal manifestations were associated with higher severity probability (OR = 2.992; 95%CI: 1.811–4.943; *p* < 0.001) (Fig. 2).

### Neuroimaging Data

**Fig. 3:**
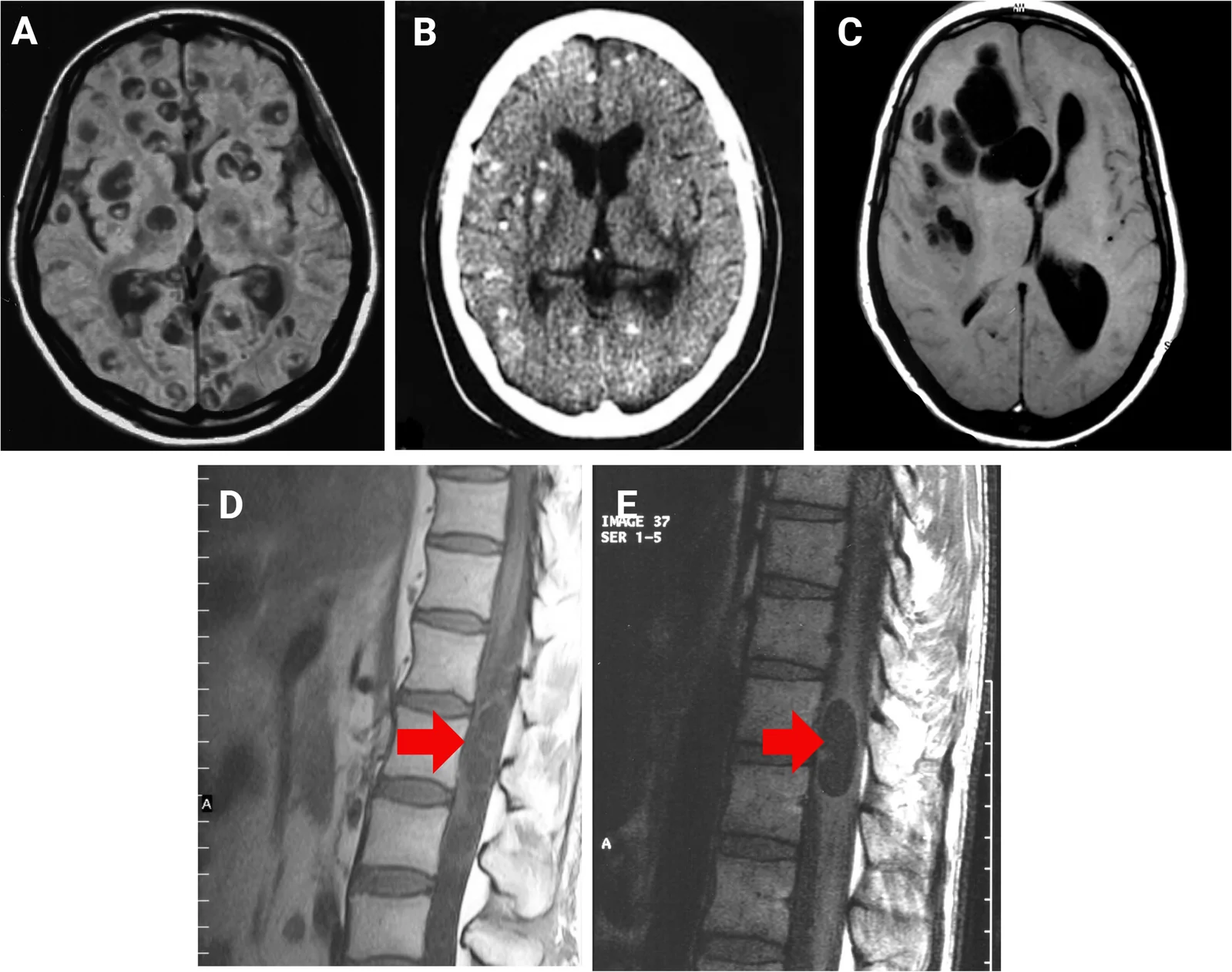
Radiological presentations of neurocysticercosis. (**A**) Brain MRI in FLAIR (fluid-attenuated inversion recovery) sequence demonstrating multiple viable cysticerci with visible scolex diffusely distributed throughout the cerebral parenchyma. (**B**) Contrast-enhanced cranial computed tomography showing the chronic phase of neurocysticercosis with multiple calcified cysticerci. (**C**) Cranial MRI in T1-weighted sequence demonstrating giant neurocysticercosis cysts. (**D**) Lumbosacral spine MRI showing an extraparenchymal cyst located within the cauda equina. (**E**) Thoracic spine MRI in T1-weighted sequence demonstrating an intramedullary cyst in the thoracic spinal cord

Magnetic resonance imaging access increased progressively in the study region (42.0% in 1991–2000 to 75.0% in 2011–2020), particularly in ISH forms (46.7% to 90.0%), while surrounding areas showed peak utilization in the intermediate period (87.5% in 2001–2010) with subsequent stabilization. Magnetic resonance imaging predominated in severe cases (77.2%) versus computed tomography (23.5%), with higher frequency of viable cysts in ISH forms (77.2% versus 32.2%) and calcifications in other clinical presentations (61.2% versus 27.2%) (Fig. 3A and B).

In 24 individuals (5.4%), NCC diagnosis was made through incidental neuroimaging findings, with giant cysts identified in 2 cases requiring neurosurgical resection (Fig. 3C). Hydrocephalus was identified in 75 patients (100% in ISH), representing 16.9% of the total sample. Spinal cysts were found in 8 patients, with 3 from other clinical presentations and 5 from ISH forms, described as 7 cysts in the spinal canal and 1 intramedullary cyst (Fig. 3D and F).

Cyst location analysis revealed 362 patients (81.7%) with isolated locations without overlap: 274 cases with purely intraparenchymal location and 88 cases with exclusively extraparenchymal location. There was frequent association of cysts in different locations in the same individual, corresponding to 81 cases (18.3%). In other clinical presentations, there was predominance of non-viable intraparenchymal cysts (74.8% of 258 analyzed), with only 2 cases having extraparenchymal subarachnoid location. As expected, our findings corroborated a strong association between severe disease and extraparenchymal cyst location, whereas intraparenchymal location predominated in the non-severe, predominantly epileptic, group. 

### Cerebrospinal fluid findings

Eighty-two patients (18.5%) presented pleocytosis, with 49 (59.7%) from the ISH form group and 33 (40.3%) from the other clinical presentations group. Cell counts ranged from 10.0 to 4,560.0/mm³, all with lymphomonocytic predominance. Significant association between pleocytosis and disease severity occurred in all analyzed regions, with stronger association in the primary study area and distant referrals (*p* < 0.001) compared to surrounding Regional Health Department XIII municipalities (*p* ≈ 0.035).

Positive cerebrospinal fluid ELISA results were obtained in 33 patients (7.4%), with 21 (63.6%) from the ISH form group and 13 (36.4%) from other clinical presentations group. In 4 cases (12.1%), titers exceeded 1/4000.

### Treatments performed

Antiparasitic treatment was performed for patients with viable intraparenchymal cysts and extraparenchymal forms when surgical approach was not indicated. Of treated intraparenchymal NCC cases, 90% received albendazole and dexamethasone in single-course treatment lasting 8 days, at albendazole dose of 15 mg/kg/day and dexamethasone dose of 10-16 mg daily. Some patients (6%) received 2 treatment courses, and 4% received 3 or more courses. For extraparenchymal cases with surgical contraindication, treatment with albendazole and dexamethasone was indicated in 62%, generally with treatment courses of 15–30 days at 15 or 30 mg/kg/day albendazole dose. Only 2 cases of extraparenchymal NCC underwent retreatment with praziquantel at 50 mg/kg/day, also associated with dexamethasone at 16 mg daily.

Therapeutic management differed significantly between groups, with greater corticosteroid use (5.1% vs. 0.6%) and methotrexate use (3.7% vs. 0%) in ISH forms, reflecting the need for more intensive interventions. The corticosteroid use specified refers to controlling symptoms secondary to chronic meningitis and refractory epileptic seizures, distinct from association with antiparasitic treatment mentioned above.

### Surgical procedures and clinical evolution

**Fig. 4:**
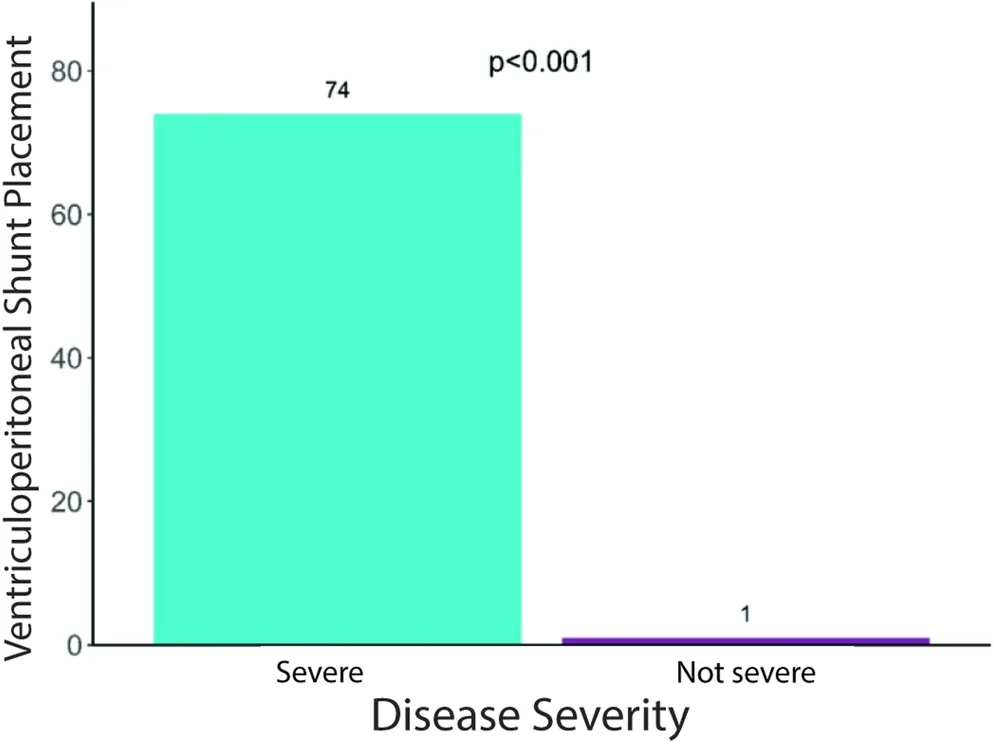
Distribution of ventriculoperitoneal shunt (DVP) procedures according to the presence of intracranial hypertension syndrome (IHS). Among 75 patients (16.9% of the total sample) who underwent DVP placement, there was an almost exclusive association with IHS, with 74 cases in the IHS group versus only 1 case in the group of other clinical presentations. This finding underscores the strong relationship between IHS (the defining criterion for the group previously labeled as severe) and the need for cerebrospinal fluid diversion

**Fig. 5:**
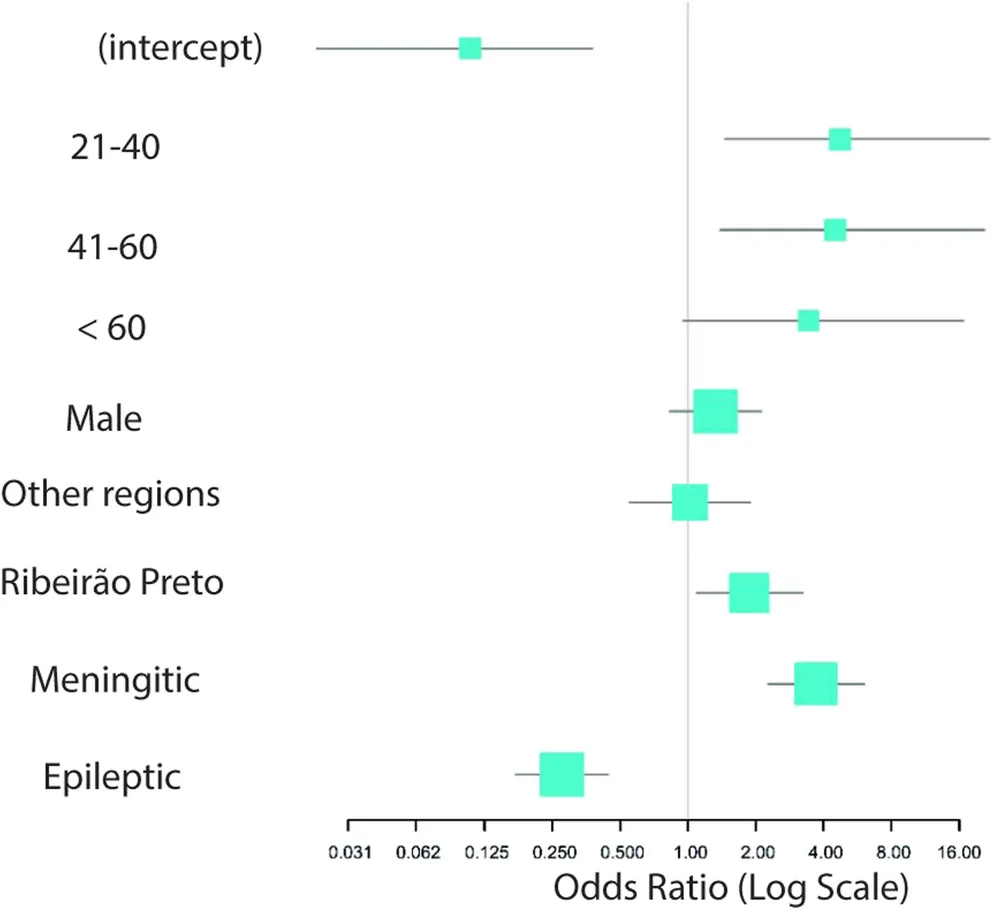
Multivariable logistic regression analysis - factors associated with severe disease. Forest plot showing odds ratios (OR) and 95% confidence intervals (CI) from multivariable logistic regression analysis identifying factors independently associated with severe neurocysticercosis. Protective factors against IHS included epileptic manifestations (OR = 0.295; 95%CI: 0.182–0.477; *p* < 0.001) and asymptomatic presentation (OR = 0.212; 95%CI: 0.111–0.404; *p* < 0.001). Meningeal manifestations were associated with increased risk of severe disease (OR = 2.992; 95%CI: 1.811–4.943; *p* < 0.001). Age groups 21–40 and 41–60 years showed increased probability of ISH forms compared to reference categories

Thirty-four patients (7.6%) underwent neurosurgery for cyst removal, with 27 (79.4%) in the ISH group, while 75 patients (16.9%) underwent ventriculoperitoneal shunt placement (Figs. 4 and 5). Notably, 16 cases underwent neurosurgical approach for cyst removal and ventriculoperitoneal shunt placement simultaneously. Surgical procedures occurred predominantly in patients from the primary study region (73.5% of surgical cases, *p* < 0.01).

Regarding hospitalizations, 160 patients (36.1%) required hospitalization, with proportional increase from 19.4% to 34.2% throughout the years despite overall case reduction. The most significant complication in the ISH group was vasculitis (2.9%), indicating strong association with disease severity, while myelitis and encephalitis complications showed no significant differences between groups. Additionally, 7 patients presented muscular forms concomitant with CNS involvement (3 other clinical presentations, 4 ISH), 2 cases of optic neuritis, and 4 cases of ventriculitis (all associated with surgical manipulation).

Among 443 patients included in detailed analysis, 63 (14.2%) died, though reliable information about death causes was unavailable, preventing definitive conclusions about NCC-death relationships. Of the total patients, 38 (8.5%) remain under follow-up, 44 (10%) were lost to follow-up, and 57 (12.8%) were discharged for secondary-level care. Nine patients (2%) had definitive diagnosis through brain biopsy for diagnostic confirmation and exclusion of differential diagnoses.

### Logistic regression analysis

Age groups demonstrated significant impact on severe disease development, with individuals aged 21–40 years showing increased ISH form probability (β = 1.55345; *p* = 0.02031) and those between 41 and 60 years similarly affected (β = 1.50821; *p* = 0.02456). For individuals over 60 years, the effect was positive but with lower statistical significance. Sex showed no statistically significant association, suggesting men and women have similar risks for severe disease.

Meningitis presence was a significant predictor, substantially increasing severe disease probability (β = 1.30861; *p* = 2.00e-07), while epilepsy presence showed a protective negative effect, significantly reducing severe disease occurrence probability (β = -1.28491; *p* = 1.02e-07).

## Discussion

This study demonstrated significant NCC incidence coefficient decline from 1995 to 2024. This epidemiological phenomenon reflects increased per capita income, improved socioeconomic and cultural levels, earlier taeniasis treatment, better sanitation conditions, greater treated water supply coverage, sewage network expansion, and stricter food quality oversight. The decline certainly reflects the local Taeniasis/Cysticercosis Prevention Program success, as incidence coefficient decline was more pronounced in the primary study region compared to other Regional Health Department XIII municipalities.

This study analyzed three decades encompassing neuroimaging examination advent and improvement, allowing more precise and early diagnosis. New therapeutic resources emerged (parasiticidal drugs, minimally invasive neurosurgical intervention), and intensive care therapy improved. These technological advances occurred alongside major basic sanitation improvements, socioeconomic-educational advancement, greater medical-hospital care access through public health system, and stricter food quality Control.

The 1993 International Task Force for Disease Eradication considered cysticercosis potentially eradicable with available resources. *Taenia solium* infection disappearance from Western European countries during the 20th century demonstrates this possibility [28]. Control required several decades and many economic, educational, and sanitary standard changes plus medical and veterinary service improvements [29, 30]. This high-incidence region pioneered mandatory notification implementation in Brazil in 1992. Despite frequency and morbidity, cysticercosis is not mandatory notifiable throughout the country, being implemented only in few localities. The decrease in new NCC case frequency was verified by disease form incidence rate reduction presenting viable cysts. Decline was observed with greater intensity in the primary study region, corroborating Prevention Program success implemented exclusively there.

The interpretation of these epidemiological trends must account for the inherent diagnostic challenges and the complex natural history of *T. solium* infection. Since NCC often presents a prolonged latent period, with larvae remaining asymptomatic for years or even decades (10–25 years), the ‘incidence’ reported in our study primarily reflects a case-notification rate. This lag between infection and symptom onset means that many cases diagnosed today represent transmission events from previous decades. Furthermore, diagnostic barriers, such as the high cost and limited availability of high-resolution neuroimaging and the low sensitivity of serological tests in cases with calcified or few lesions, contribute to this ‘delayed’ detection. This phenomenon explains the observed ‘catch-up’ diagnostic effect, where improved access to tertiary care and advanced imaging reveals a pool of long-standing, often severe, infections that had remained hidden within the community.

This analysis of 465 patients revealed 29.2% prevalence of IHS, aligning with international literature reporting frequencies between 11.7% and 30% [31]. Results demonstrated significant intracranial hypertension syndrome frequency decrease from 1996 to 2024. Comparing slope lines of both clinical presentations, the decline of other clinical forms was more pronounced than the decline of the IHS form, a trend plausibly reflecting the combined effect of improved public health measures targeting the taeniasis–cysticercosis complex, earlier etiological diagnosis, wider availability of antiparasitic therapy, and progressively greater access to neuroimaging, allowing clinical intervention before progression to advanced disease stages associated with raised intracranial pressure.

Clinical manifestation profile demonstrated significant association between intracranial hypertension syndrome and meningeal manifestations (47.7% in ISH form group), corroborating literature data [32], which is pathophysiologically consistent with extraparenchymal meningeal involvement, where chronic inflammation, arachnoiditis, and basal cisternal disease impair cerebrospinal fluid circulation through inflammatory and obstructive mechanisms, ultimately leading to hydrocephalus and intracranial hypertension.

Epileptic manifestation was significantly more prevalent in non-severe group, also supporting previous findings [33], reflecting the predominance of intraparenchymal cysts, isolated granulomas, and calcified lesions in these patients, which constitute structurally epileptogenic substrates but are less likely to disrupt cerebrospinal fluid dynamics or cause diffuse inflammatory obstruction.

Neuroimaging findings demonstrated significant viable cyst predominance in ISH form (77.2% vs 30.1%), consistent with current guidelines emphasizing this association particularly in extraparenchymal locations. Magnetic resonance imaging access evolution reflects progressive adaptation to technical recommendations, likely contributing to improved detection of viable and extraparenchymal cysts that may have been underdiagnosed in earlier decades due to limited imaging availability. As suggested by the rising proportion of severe cases despite declining incidence, this trend likely reflects a ‘catch-up’ diagnostic effect. Given that racemose and extraparenchymal NCC can remain asymptomatic for 10–25 years, the improved access to high-resolution MRI has facilitated the identification of long-standing, chronic infections that present with intracranial hypertension rather than early-onset seizures, which may have been underdiagnosed in earlier decades.

Current management of intraparenchymal NCC has evolved towards more potent antiparasitic regimens. International guidelines, such as those from the IDSA/ASTMH, now recommend combined therapy with albendazole and praziquantel for patients with more than two viable parenchymal cysts, as this combination has proven superior to albendazole monotherapy in achieving complete cyst resolution [34–36]. Recent prospective data further support this approach, demonstrating that dual therapy can achieve cyst resolution rates as high as 95%, significantly outperforming the rates observed with monotherapy [35]. Although our cohort primarily received albendazole monotherapy (90% of cases), reflecting the standard practice during the majority of this 30-year study period, these emerging data underscore the importance of considering combined treatment to optimize parasite clearance in parenchymal disease [37, 38]. Extraparenchymal cyst treatment with surgical contraindication was performed with albendazole and dexamethasone, following current NCC management guidelines [36]. Recent comprehensive reviews highlight the persistent controversy over parasitic therapy validity, emphasizing the need for additional randomized controlled trials with long-term clinical outcome evaluation [37].

Neurosurgical approach was indicated for cyst removal in 7.6% and ventriculoperitoneal shunt placement in 16.9% of patients, with tendency toward procedure reduction over years, a finding likely attributable to earlier diagnosis, optimization of medical and anti-inflammatory management, and increasing use of minimally invasive endoscopic techniques, collectively reducing the need for aggressive surgical interventions and permanent cerebrospinal fluid diversion.

Most neurosurgical cases underwent endoscopic approach for intraventricular cyst excision, consistent with literature data [38].

This study presents limitations inherent to retrospective design. Evolution in diagnostic criteria and imaging method availability over three decades may have introduced detection bias. Furthermore, the diagnostic challenges highlighted in our 30-year cohort are compounded globally by the lack of reliable point-of-care (POC) tests. As noted in recent literature [39], the absence of such tools in primary care settings hinders the identification of individuals who require neuroimaging, often resulting in late-stage diagnoses when symptoms are already severe. This diagnostic gap is particularly critical because international guidelines, such as those from the IDSA/ASTMH [36], still rely heavily on high-resolution neuroimaging (CT and MRI) and specialized serology for definitive classification and treatment planning, resources that are often unavailable in many endemic regions. Therefore, the development and implementation of antigenic-based POC tests are urgent. These tools would not only facilitate earlier diagnosis, potentially before symptom onset, but also ensure that scarce neuroimaging resources are prioritized effectively, preventing patients from undergoing unnecessary treatments or suffering from delayed interventions. Sample size reduction in recent periods limits statistical power for stratified analyses. Severity definition based exclusively on intracranial hypertension manifestation may have underestimated other clinically relevant presentations.

The methodology presents robust aspects strengthening findings validity. Temporal analysis spanning three decades allows unprecedented evaluation of NCC severity profile evolution in a Brazilian tertiary center. The study provides significant contributions to current NCC knowledge, with detailed characterization of ISH form temporal evolution filling important gaps in Latin American literature.

## Conclusions

This three-decade analysis demonstrated significant NCC incidence decline in this endemic region, with more pronounced reduction compared to surrounding regions, supporting mandatory notification and prevention program effectiveness. Progressive increase in the proportion of IHS cases, despite overall incidence decline, suggests improved detection capabilities and diagnostic standardization.

Clinical manifestation patterns confirmed literature findings, with epileptic forms predominating in non-severe cases and meningeal manifestations associating with severity. Multivariable analysis identified age, epileptic manifestations, and meningeal presentations as independent factors associated with disease severity.

Neuroimaging evolution, particularly magnetic resonance imaging (MRI) access improvement, contributed to better ISH form detection. In extraparenchymal cases due to hydrocephalous condition MRI are inconclusive so there is an urgent need of a point care test for neurocysticercosis. Treatment patterns aligned with current international guidelines, with reduced surgical interventions over time reflecting conservative management advances.

These findings support continued mandatory notification implementation and prevention program expansion to other endemic regions. Future research should focus on long-term patient outcomes and prevention program cost-effectiveness evaluation. *T. solium* Antigenic based confirmation tests should be developed so that patient will not suffer from irrelevant cysticercidal therapy and disability adjusted years in life. 

## Data Availability

Anonymized data will be made available to qualified investigators upon reasonable request to the corresponding author.

## Abbreviations

ALB: Albendazole
CI: Confidence Interval
CNS: Central Nervous System
CSF,: Cerebrospinal Fluid
CT: Computed Tomography
DRS XIII: Regional Health Department XIII
DVP/VPS: Ventriculoperitoneal Shunt
EITB: Enzyme-linked Immunotransfer Blot
ELISA: Enzyme-Linked Immunosorbent Assay
HCFMRP-USP: Clinical Hospital of Ribeirão Preto School of Medicine, University of São Paulo
MRI: Magnetic Resonance Imaging
NCC: Neurocysticercosis
OR: Odds Ratio
PCR: Polymerase Chain Reaction
PZQ: Praziquantel
SUS: Sistema Único de Saúde (Brazilian Public Health System)
WHO: World Health Organization
